# Regulatory T cell therapy in autoimmune and immune-mediated diseases: from basic research to clinical practice and future perspectives

**DOI:** 10.3389/fimmu.2026.1772001

**Published:** 2026-05-07

**Authors:** Shihao Duan, Junyan Zhang, Zhongxiu Chen, Yong He, Yingzhi Zhang, Hong Guo

**Affiliations:** 1 Department of Gastroenterology, Chongqing Academy of Medical Sciences, Chongqing General Hospital, Chongqing University, Chongqing, China; 2 Department of Cardiology, West China Hospital of Sichuan University, Chengdu, China

**Keywords:** regulatory T cells, autoimmune diseases, immune-mediated diseases, adoptive cell therapy, CAR-Treg

## Abstract

Regulatory T cells (Tregs) are pivotal immune modulators essential for maintaining immune homeostasis and preventing aberrant immune responses. In recent years, Treg-based therapies have emerged as a promising strategy for treating a variety of non-malignant diseases, including autoimmune disorders, transplantation-related complications, and allergic conditions. This review provides a comprehensive overview of the discovery and evolution of Tregs, detailing their immunoregulatory mechanisms that underpin their therapeutic potential. We systematically evaluate current clinical applications of Treg therapy in diverse non-tumor pathologies, highlighting both the efficacy and safety outcomes reported in ongoing clinical trials. Additionally, the review addresses the challenges faced in translating Treg therapies from bench to bedside, such as cell stability, expansion methodologies, and functional heterogeneity. Finally, we explore future directions in Treg research, including innovative therapeutic approaches, advances in gene engineering technologies, and improvements in cell expansion techniques, all aimed at enhancing the clinical translation and therapeutic efficacy of Treg-based interventions. This article aims to provide a thorough theoretical foundation and practical guidance to advance the application of Treg therapy in non-malignant diseases.

## 1 Introduction

Regulatory T cells (Tregs) constitute a specialized subset of CD4+ T lymphocytes characterized primarily by the expression of the transcription factor forkhead box protein P3 (Foxp3), which is indispensable for their development and suppressive function. These cells serve as pivotal mediators of immune homeostasis, orchestrating the balance between immune activation and tolerance to self-antigens, thereby preventing the onset of autoimmune diseases and maintaining peripheral tolerance. The immunosuppressive capacity of Tregs extends beyond the containment of autoreactive T cells; they also play crucial roles in modulating immune responses to commensal microbiota, allergens, and tissue-specific antigens, ensuring the fine-tuning of immune reactivity in diverse physiological contexts (1, 2). The significance of Tregs in immune regulation is underscored by the observation that their quantitative or functional deficiencies are implicated in a broad spectrum of immunopathologies, including autoimmune disorders, allergic diseases, and chronic inflammatory conditions. In this light, Tregs have emerged as a promising cellular target for therapeutic interventions aimed at restoring immune tolerance without compromising global immune competence.

The pathogenesis of non-tumor diseases such as autoimmune disorders, allergic inflammation, and chronic inflammatory diseases is often characterized by a breakdown in immune tolerance mechanisms, leading to aberrant activation of effector immune cells against self or innocuous antigens. This immune dysregulation is frequently associated with defects in Treg number, phenotype, or suppressive function, which collectively contribute to the perpetuation of pathological immune responses (3, 4). For instance, in autoimmune diseases like type 1 diabetes (T1D), rheumatoid arthritis (RA), and systemic lupus erythematosus (SLE), the failure to adequately control autoreactive T cells by Tregs results in tissue-specific damage and chronic inflammation (5, 6). Similarly, in allergic diseases, subversion of Treg function leads to unrestrained type 2 immune responses and tissue pathology (5). These insights highlight the therapeutic potential of strategies aimed at enhancing Treg-mediated immune regulation to re-establish tolerance and ameliorate disease.

Recent years have witnessed remarkable advancements in the understanding of Treg biology, from their molecular development and tissue-specific adaptations to their functional specialization in various non-lymphoid organs. Tissue-resident Tregs exhibit distinct transcriptional profiles and antigen specificities that enable them to maintain local immune homeostasis and contribute to tissue repair and regeneration beyond their canonical immunosuppressive roles (7, 8). Moreover, innovative approaches to modulate or harness Tregs, including ex vivo expansion, induction of antigen-specific Tregs, and pharmacological conversion of conventional T cells into Tregs, have shown promising results in preclinical models and early-phase clinical trials targeting non-tumor diseases (9, 10). For example, immune-homeostatic microparticles designed to induce apoptosis of activated T cells and promote Treg differentiation have demonstrated efficacy in mouse models of autoimmunity (11). Additionally, novel small molecules that convert memory CD4+ T cells into suppressive Foxp3+ Tregs via modulation of signaling pathways offer potential for inducing immune tolerance (11). These therapeutic innovations underscore the translational momentum from foundational immunology towards clinical application.

Given the expanding landscape of Treg research and its translational implications, there is an urgent need for comprehensive and systematic reviews that integrate the latest biological insights, therapeutic strategies, and clinical outcomes related to Treg therapy in non-tumor diseases. Such analyses are critical to delineate the current state of the art, identify challenges such as Treg stability, antigen specificity, and functional heterogeneity, and forecast future directions including combination therapies and precision immunomodulation. This review aims to provide an exhaustive synthesis of the biological characteristics of Tregs, their immunoregulatory mechanisms, and their clinical applications in non-tumor diseases. By evaluating existing clinical trial data and emerging therapeutic modalities, we seek to offer a nuanced perspective on the prospects of Treg-based interventions and outline strategic avenues for enhancing their efficacy and safety in diverse pathological contexts.

## 2 Regulatory T cells: biology, mechanisms, and therapeutic applications

### 2.1 Biological characteristics of treg cells

#### 2.1.1 Historical milestones

The discovery of regulatory T (Treg) cells marked a pivotal moment in immunology, fundamentally reshaping our understanding of immune tolerance and autoimmunity. Initially identified as a subset of CD4+ T cells expressing high levels of CD25, the interleukin-2 receptor alpha chain, Tregs were characterized by their unique ability to suppress immune responses and maintain self-tolerance. The landmark identification of the transcription factor Foxp3 as a lineage-defining marker of Treg cells was crucial, as Foxp3 expression was shown to be indispensable for Treg development and function. Early experimental work demonstrated that mutations in Foxp3 led to severe autoimmune syndromes, underscoring the essential role of Tregs in immune regulation. Key experiments, such as adoptive transfer studies in mice, established that Tregs could prevent autoimmune disease by suppressing autoreactive T cells. These foundational studies firmly positioned Tregs as central players in immune homeostasis, highlighting their suppressive capacity through cell contact-dependent mechanisms and cytokine secretion. The identification of surface markers CD4, CD25, and intracellular Foxp3 provided tools for isolating and studying Tregs, facilitating subsequent research into their biology and therapeutic potential. More recently, the field has undergone a conceptual shift from viewing Tregs solely as immunosuppressive cells to recognizing them as tissue-integral regulators with context-specific functions extending far beyond canonical immune suppression, including roles in tissue repair, metabolic homeostasis, and stem cell niche maintenance (12, 13). Collectively, these milestones laid the groundwork for recognizing Treg cells as the core regulators of immune tolerance and opened avenues for their clinical application in autoimmunity, transplantation, and beyond (14, 15).

#### 2.1.2 Cell biology and subtype evolution

Regulatory T cells (Tregs) exhibit considerable heterogeneity, classified primarily into thymus-derived natural Tregs (nTregs or tTregs) and peripherally induced Tregs (iTregs or pTregs). nTregs develop in the thymus during T cell maturation and are characterized by stable expression of Foxp3, which is critical for their suppressive function and lineage stability. In contrast, iTregs arise from conventional CD4+ T cells in peripheral tissues upon antigenic stimulation under tolerogenic conditions, such as in the presence of transforming growth factor-beta (TGF-β). Foxp3 acts as the master transcription factor orchestrating Treg development, function, and maintenance by regulating gene expression programs that confer suppressive capacity. The expression of Foxp3 is tightly controlled by epigenetic modifications and post-translational modifications, ensuring Treg lineage stability. The surface markers, tissue distribution patterns, and functional properties of major Treg subsets are summarized in **Table 1**.

**Table 1:** Summary of major Treg populations: markers, tissue distribution, and functional features.

Treg subset	Core markers	Additional key markers	Primary tissue location	Key functional features
Classification by developmental origin				
Thymic Tregs (tTregs/nTregs)	CD4^+^CD25^+^Foxp3^+^	Helios^+^ (enriched), Nrp-1^+^ (in mice), TSDR demethylated	Thymus, secondary lymphoid organs	Stable suppressive function; self-antigen tolerance; stable Foxp3 expression
Induced Tregs (iTregs/pTregs)	CD4^+^CD25^+^Foxp3^+^	Helios^-^/low (enriched), Nrp-1^-^/low (in mice)	Peripheral tissues, mucosal sites	Induced by TGF-β in periphery; tolerance to non-self antigens including commensal microbiota and dietary antigens
Classification by activation/functional state				
Central Tregs (cTregs)	CD4^+^CD25^+^Foxp3^+^	CCR7^+^, CD62L^+^, CD44low	Secondary lymphoid organs (lymph nodes, spleen)	Quiescent state; lymphoid tissue homing; IL-2 dependent survival
Effector Tregs (eTregs)	CD4^+^CD25^+^Foxp3^+^	CD44hi, CD62Llow, ICOS^+^, KLRG1^+^	Non-lymphoid peripheral tissues	Enhanced suppressive capacity; tissue migration; activated phenotype
Classification by transcriptional specialization				
Th1/Th2/Th17-like Tregs	CD4^+^CD25^+^Foxp3^+^	T-bet^+^/CXCR3^+^ (Th1-like); IRF4^+^/GATA3^+^ (Th2-like); RORγt^+^/CCR6^+^ (Th17-like)	Sites corresponding to respective Th inflammation	Co-expression of lineage-defining transcription factors; preferential suppression of matched Th responses
Classification by anatomical location				
Colonic/Intestinal Tregs	CD4^+^CD25^+^Foxp3^+^	RORγt^+^ (subset), IL-10^+^	Colon, small intestine lamina propria	Microbiota-induced tolerance; mucosal homeostasis
Follicular Regulatory T cells (Tfr)	CD4^+^CD25^+^Foxp3^+^	Bcl-6^+^, CXCR5^+^, PD-1^+^	Germinal centers, B cell follicles	Regulation of germinal center reactions; control of Tfh and B cell responses

Bcl-6, B-cell lymphoma 6; CCR6, C-C chemokine receptor type 6; CCR7, C-C chemokine receptor type 7; CD, cluster of differentiation; CD62L, L-selectin; cTreg, central regulatory T cell; CXCR3, C-X-C motif chemokine receptor 3; CXCR5, C-X-C motif chemokine receptor 5; eTreg, effector regulatory T cell; Foxp3, forkhead box P3; GATA3, GATA binding protein 3; ICOS, inducible T cell costimulator; IL-2, interleukin-2; IL-10, interleukin-10; IRF4, interferon regulatory factor 4; iTreg, induced regulatory T cell; KLRG1, killer cell lectin-like receptor G1; Nrp-1, neuropilin-1; nTreg, natural regulatory T cell; PD-1, programmed cell death protein 1; pTreg, peripheral regulatory T cell; RORγt, retinoic acid receptor-related orphan receptor gamma t; T-bet, T-box transcription factor TBX21; Tfh, T follicular helper cell; Tfr, follicular regulatory T cell; TGF-β, transforming growth factor-beta; Th, T helper; TSDR, Treg-specific demethylated region; tTreg, thymic regulatory T cell.

The concept of “tissue adaptation” has emerged as a central paradigm: upon entry into non-lymphoid tissues, Tregs undergo transcriptional reprogramming driven by tissue-specific transcription factors (e.g., peroxisome proliferator-activated receptor gamma (PPARγ) in adipose tissue, B lymphocyte-induced maturation protein 1 (Blimp-1) in barrier tissues, cellular musculoaponeurotic fibrosarcoma oncogene homolog (c-Maf) in gut), local metabolites (short-chain fatty acids, retinoic acid, bile acids), and stromal cell-derived signals (16, 17). This adaptation involves not only acquisition of tissue-homing receptors but also fundamental rewiring of metabolic and effector programs. Notably, tissue Tregs often exhibit reduced TCR signaling requirements for maintenance compared to lymphoid Tregs, instead relying more heavily on cytokine signals, such as interleukin (IL)-2 and IL-33, as well as metabolic inputs (18, 19). Different Treg subtypes display tissue-specific distribution and functional specialization; for instance, tissue-resident Tregs in non-lymphoid organs like adipose tissue, skin, and intestine exhibit unique transcriptomic profiles and contribute to local homeostasis beyond immune suppression, including tissue repair and metabolic regulation. Moreover, Treg subsets vary in their expression of surface molecules and cytokine profiles, reflecting their adaptability to diverse immunological contexts. The dynamic interplay between nTregs and iTregs, along with their phenotypic and functional plasticity, underlies the complexity of immune regulation mediated by Tregs in health and disease (15, 20, 21).

#### 2.1.3 Mechanisms of action

**Figure 1:**
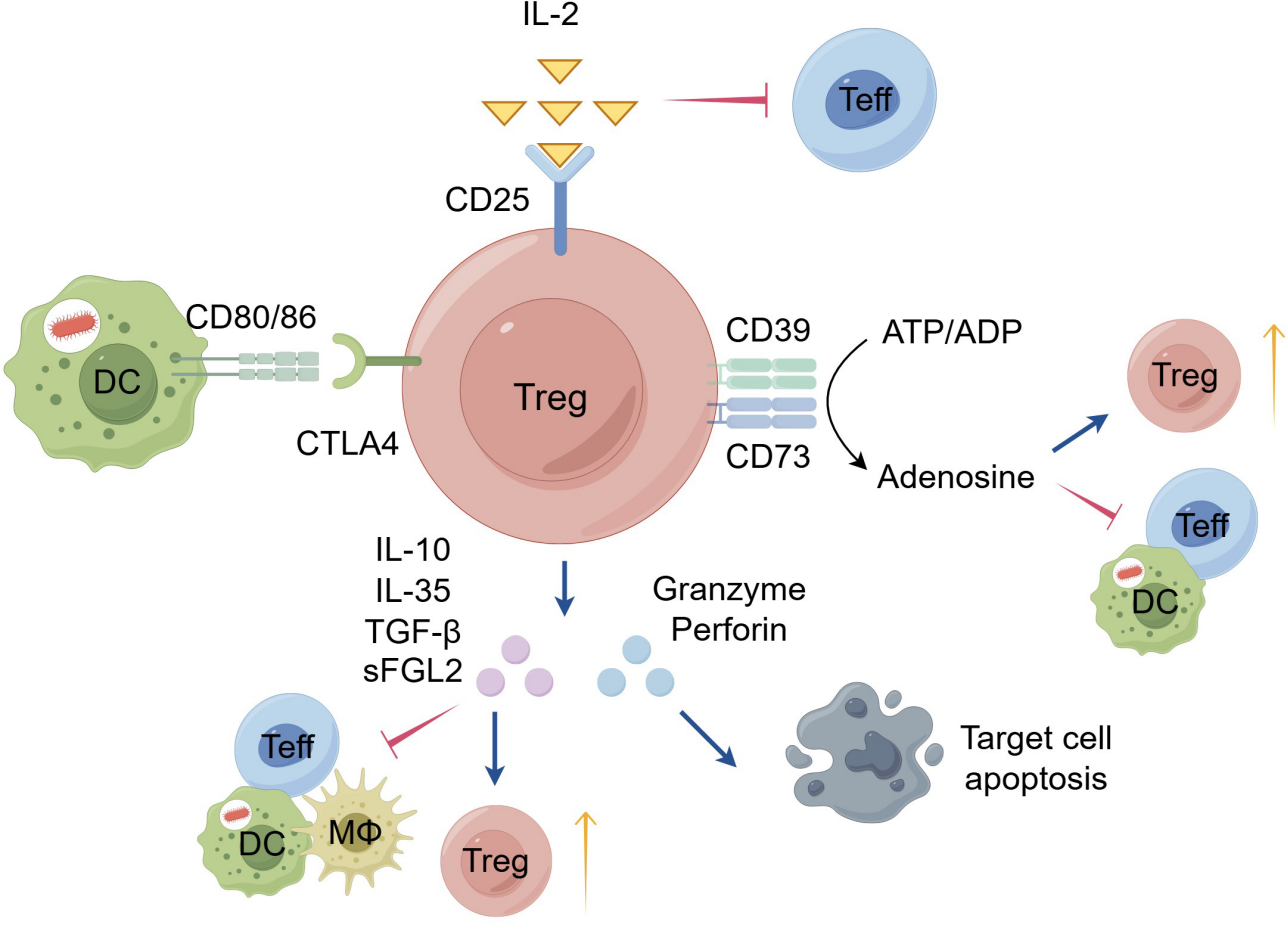
Suppressive mechanisms of Tregs. Tregs mainly exert immunosuppressive effects through the following four mechanisms: 1) Secretion of inhibitory cytokines, including IL-10, IL-35, TGF-β and FGL2, which suppress the proliferation and function of effector T cells and antigen-presenting cells, while also promoting the expansion of Treg cells. 2) Engagement of inhibitory receptors, whereby Tregs constitutively express CTLA-4 that binds to CD80/CD86 molecules on antigen-presenting cells, competitively blocking the co-stimulatory signals required for effector T cell activation. 3) Metabolic interference: On one hand, Tregs constitutively express high levels of the IL-2 receptor α chain (CD25), enabling them to consume abundant IL-2 from the local microenvironment and thereby restrict effector T cell expansion; on the other hand, Tregs express extracellular nucleotidases CD39 and CD73 on their surface, which progressively hydrolyze immune-activating ATP and ADP into adenosine, a potent immunosuppressive metabolite. 4) Induction of cell apoptosis, through which Tregs directly induce the apoptosis of activated effector T cells or antigen-presenting cells by secreting granzymes and perforin. CTLA-4, cytotoxic T lymphocyte-associated antigen-4; DC, dendritic cell; Treg, regulatory T cell; MΦ, macrophage; Teff, effector T cells; IL, Interleukin; TGF-β, transforming growth factor-beta; sFGL2, soluble fibrinogen like protein 2; ATP, adenosine triphosphate; ADP, adenosine diphosphate. (This figure was drawn by Figdraw).

Tregs employ a context-dependent repertoire of suppressive mechanisms to maintain immune homeostasis (**Figure 1**). Core pathways include metabolic control through high CD25 expression, enabling IL-2 sequestration and limiting conventional T cell proliferation in environments where IL-2 availability fluctuates; cytotoxic T lymphocyte-associated antigen (CTLA-4)–mediated suppression that reprograms antigen-presenting cells to dampen costimulation; and ectonucleotidase activity via CD39 and CD73 that convert pro-inflammatory ATP to anti-inflammatory adenosine, shaping a tolerogenic milieu in barrier tissues such as gut and skin (22, 23). Additional soluble mediators—TGF-β, IL-35, IL-10, and fibrinogen like protein 2 (FGL2)—broadly dampen effector functions and promote regulatory phenotypes. Tregs also possess granzyme–perforin–dependent cytotoxicity that can selectively eliminate overactive or cytotoxic targets, contributing to tolerance in transplantation and autoimmunity (24). Importantly, these mechanisms show tissue- and state-specific emphasis: in the gut, TGF-β and retinoic acid foster iTreg induction and stability with CD39/CD73-adenosine signaling playing a pivotal anti-inflammatory role; in adipose tissue, IL-10 and metabolic adaptations enable suppression of pro-inflammatory macrophages and maintenance of insulin sensitivity; in barrier organs, Tregs cooperate with local cues to sustain barrier integrity and promote repair; and in inflamed joints, CTLA-4 and IL-2–driven expansion must contend with inflammatory milieus that can undermine FOXP3 stability and functional capacity (25, 26). In autoimmune inflammation, persistent inflammatory cues erode this regulatory network: pro-inflammatory cytokines (e.g., IL-6, IL-1β, TNF-α) destabilize FOXP3 signaling, enhanced APC costimulation heights Teff reactivity, and tissue-specific constraints create niches where suppression falters (23, 27–30). These dynamics explain why Treg-based therapies may require organ- or disease-specific tuning and combination strategies, including antigen-specific Tregs and localized delivery, to sustain tolerance. A further dimension is Treg stability and lineage plasticity: inflammatory milieus can destabilize FOXP3 via epigenetic and metabolic perturbations, driving ex-Tregs or Th17-like conversions that compromise tolerance. Therapeutically, stabilizing FOXP3 through sustained IL-2 signaling, metabolic modulation, and epigenetic targeting—alongside engineering approaches such as CAR- or TCR-Tregs with built-in stability safeguards—constitutes a critical priority. In translational terms, these insights imply that Treg products should be tailored to tissue context, disease stage, and concomitant inflammatory pathways, with combination regimens and robust immunomonitoring to guide dosing and delivery.

#### 2.1.4 Stability and lineage plasticity in inflammation

Treg stability and lineage plasticity are central to the success of Treg-based therapies in autoimmune and inflammatory diseases (31). Inflammatory cues can destabilize FOXP3 expression through epigenetic and transcriptional mechanisms, promoting ex-Tregs or Th17-like reprogramming with direct implications for clinical outcomes. FOXP3 stability relies on TSDR demethylation and sustained FOXP3 transcriptional networks driven by IL-2–STAT5 signaling; inflammatory signals such as IL-6–STAT3 and IL-1β can shift transcriptional programs toward Th17 features, while metabolic reprogramming from oxidative phosphorylation toward glycolysis further undermines regulatory identity (32, 33). Consequently, biomarkers of stability (FOXP3 levels, TSDR methylation, metabolic state, cytokine signaling profiles) become essential for patient stratification and real-time monitoring (34). Therapeutic strategies to bolster stability include persistent low-dose IL-2 to sustain STAT5 activity, localized delivery and co-treatments that curb pro-inflammatory cues (e.g., retinoic acid signaling, TGF-β–mediated regulation), and epigenetic or metabolic interventions to preserve TSDR demethylation and regulatory metabolism (35). Engineered Tregs also require designs that maintain FOXP3 expression in inflammatory environments and resist circuit-driven drift (36). Clinically, stability-focused regimens should be disease- and organ-specific, balancing durable tolerance with preservation of host defense, and should be coupled with adaptive immunomonitoring and flexible dosing.

### 2.2 Clinical applications of treg cell therapy

#### 2.2.1 Autoimmune diseases

**Figure 2:**
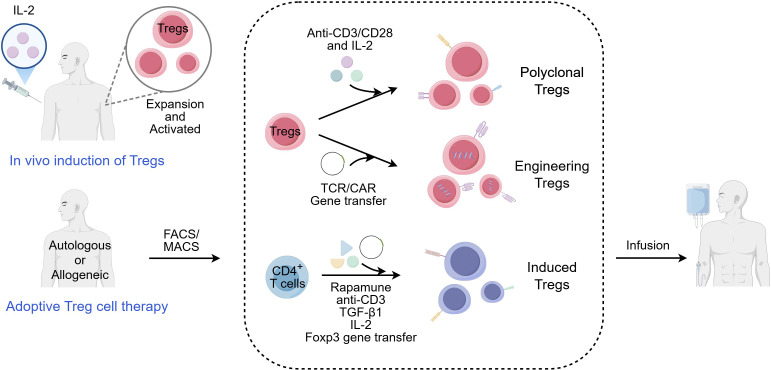
Clinical Treg treatment methods. In the current clinical trials, Treg therapy mainly involves low-dose IL-2 to induce the expansion of Tregs in vivo and adoptive Treg cell therapy. Adoptive Treg cell therapy primarily comprises three cellular sources: (1) Isolation and in vitro expansion of natural polyclonal Tregs from sources like peripheral blood; (2) Genetic engineering of Tregs to express a TCR or a CAR conferring specificity for a target antigen peptide; (3) In vitro reprogramming of conventional CD4+ T cells into Treg-like cells with regulatory function using cytokines (e.g., TGF-β, rapamycin) or genetic engineering (e.g., FOXP3 transduction). Tregs, regulatory T cells; FACS, fluorescence-activated cell sorting; MACS, magnetic-activated cell sorting; TCR, T-cell receptor; CAR, chimeric antigen receptor; IL, Interleukin; TGF-β, transforming growth factor-beta; FOXP3, forkhead box protein P3. (This figure was drawn by Figdraw).

In autoimmune diseases such as T1D, RA, and multiple sclerosis (MS), dysfunction or deficiency of Tregs contributes to the breakdown of immune tolerance, leading to tissue-specific autoimmunity (4, 37). Typical clinical applications of Treg cell therapy in autoimmune diseases are summarized in **Table 2**. The therapeutic application of Tregs aims to restore this balance by either expanding endogenous Tregs or adoptively transferring ex vivo expanded Tregs to suppress pathogenic effector T cells (38) (**Figure 2**).

**Table 2:** Typical clinical applications of regulatory T cell therapy in autoimmune diseases.

Disease category	Specific indication	Therapy type	Development stage	Key endpoints/outcomes	Major limitations	Representative trials
Type 1 Diabetes	Recent-onset T1D	Autologous polyclonal Tregs (monotherapy)	Phase I/II	Safe in children/adolescents; No significant C-peptide preservation; Inverse correlation between expansion fold and efficacy	Limited in vivo persistence; Lack of functional durability; Small sample sizes (n=12-14); Short follow-up (1-2 years)	NCT01210664; NCT02691247
	Recent-onset T1D	Tregs + anti-CD20 (Rituximab)	Phase I/II	Attenuated β-cell loss; Preserved C-peptide levels; Reduced insulin requirements; PD-1+ T cells as predictive biomarker	Combination effects difficult to dissect; Limited long-term data; Patient heterogeneity in response	NCT02772679
Systemic Lupus Erythematosus	Active SLE	Low-dose IL-2	Phase I/II	Increased Treg numbers; Reduced SLEDAI scores; Favorable safety profile; Restored Treg function	Optimal dose/duration unclear; Heterogeneous patient populations; Limited Phase III data	NCT02955615
	SLE	Novel IL-2 analogs (NKTR-358, efavaleukin alfa, MK-6194)	Phase I/II	Prolonged half-life; Enhanced Treg selectivity; Improved patient compliance	Early development stage; Long-term safety unknown; Cost considerations	NCT03556007
Multiple Sclerosis	MS (various forms)	Low-dose IL-2	Phase II (RCT)	Modest delayed Treg expansion; Elevated Treg frequencies in some patients; No significant clinical benefit	Attenuated response vs. other autoimmune diseases; Clinical outcomes not significant; Patient selection unclear	NCT02424396
Pemphigus & Autoimmune Skin Diseases	Pemphigus vulgaris	DSG3-specific Tregs	Phase I/IIa	Establishes feasibility of antigen-specific approach; Safe and well-tolerated	Early phase; Efficacy not yet demonstrated; Manufacturing complexity	NCT03239470
Inflammatory Bowel Disease	Crohn's disease	Ovalbumin-specific Tregs (CATS29)	Phase I/IIa	Safety demonstrated; Potential clinical benefit; Compatible with gut inflammation	Model antigen (not disease-relevant); Limited efficacy data; Small sample size	NCT03185000

T1D, type 1 diabetes; Tregs, regulatory T cells; SLE, systemic lupus erythematosus; IL-2, interleukin-2; SLEDAI, Systemic Lupus Erythematosus Disease Activity Index; MS, multiple sclerosis; RCT, randomized controlled trial; DSG3, desmoglein 3; PD-1, programmed cell death protein 1.

##### 2.2.1.1 T1D

In T1D, regarding Treg cell monotherapy, multiple phase 2 clinical trials have demonstrated that a single infusion of autologous polyclonally expanded Treg cells is safe in children and adolescents with recent-onset T1D, yet it failed to significantly delay β-cell functional decline (no significant difference in C-peptide level reduction) (39, 40). The limited efficacy of Treg therapies in preserving β-cell function in T1DM may reflect late intervention timing after substantial β-cell loss, and whether targeting earlier disease stages could improve outcomes warrants investigation. Additionally, the diabetogenic milieu may compromise Treg stability and function, suggesting that genetic engineering approaches to enhance FOXP3 stability or inflammatory resistance merit exploration. Furthermore, the lack of antigen specificity in polyclonal Treg products may limit therapeutic precision, raising the possibility that TCR-engineered or CAR-Treg strategies targeting islet autoantigens could offer improved efficacy, although clinical validation remains needed. Although ex vivo expanded Treg cells exhibit robust activity and suppressive capacity, their *in vivo* persistence and functionality remain limited, with expansion fold inversely correlating with therapeutic efficacy (39, 41). Regarding Treg-based combination immunotherapy, co-administration of Treg cells with anti-CD20 antibody (rituximab) significantly attenuated β-cell function loss in children with new-onset T1D, preserving C-peptide levels, reducing insulin requirements, and prolonging remission periods in a subset of patients (42, 43). PD-1-positive T cells may serve as a biomarker for predicting combination therapy efficacy (43). Furthermore, low-dose IL-2 selectively promotes *in vivo* Treg cell expansion, with high responders demonstrating superior C-peptide preservation and enhanced immunoregulatory function (44, 45). Additionally, dendritic cell-based therapies (such as AVT001) and anti-thymocyte globulin (ATG) can also delay T1D progression through modulation of the Treg/Teff balance (46–48).

##### 2.2.1.2 SLE

Patients with SLE commonly exhibit reduced Treg cell numbers and functional impairment, resulting in disrupted immune tolerance and enhanced autoimmune responses (49). Treg deficiency is closely associated with dysregulation of the IL-2 signaling pathway, and the Treg/Th17 imbalance is considered one of the central mechanisms in SLE pathogenesis (49). Low-dose IL-2 can selectively expand Tregs, restore their immunosuppressive function, and ameliorate disease activity in SLE patients (49–51). Multiple randomized controlled trials and systematic reviews have demonstrated that low-dose IL-2 therapy significantly increases Treg numbers, reduces SLEDAI scores, and exhibits favorable safety profiles (52–54). Novel IL-2 analogs (such as NKTR-358, efavaleukin alfa, and MK-6194) further enhance therapeutic efficacy and patient compliance through prolonged half-life and enhanced Treg selectivity (55, 56).

Ex vivo-expanded autologous Treg cell infusion has shown promise in ameliorating SLE manifestations in animal models and selected clinical cases, with some patients demonstrating long-term Treg survival *in vivo (*57, 58). Emerging cellular therapies, including anti-CD19 CAR-Tregs and Sm antigen-specific Tregs, have exhibited robust efficacy and safety in animal and humanized mouse models, effectively suppressing autoreactive B cells and attenuating the progression of renal injury (57, 59). Additionally, various other therapeutic modalities—including pharmacological agents, exosomes, nanoparticles, and metabolic regulation—have demonstrated potential in modulating Treg/Th17 balance, promoting Treg differentiation, and improving immune dysregulation and clinical manifestations in SLE animal models and selected patient cohorts (60–62). However, although polyclonal Tregs have demonstrated therapeutic efficacy in SLE patients, their clinical translation remains limited due to several challenges, including the large number of cells required for infusion, lack of tissue-specific targeting, and inadequate *in vivo* persistence. Moreover, to date, no clinical trials of CAR-Treg therapy for SLE have been initiated. Key bottlenecks remain, including transduction efficiency, long-term stability, safety concerns, and the actual functionality of CAR-Tregs in complex inflammatory microenvironments. Further clinical application of CAR-Tregs is eagerly anticipated.

##### 2.2.1.3 RA

Animal models and early-phase clinical studies have demonstrated that infusion of autologous or allogeneic Tregs can alleviate joint inflammation and restore immune tolerance (63, 64).

Multiple studies have confirmed that low-dose IL-2 selectively expands Tregs, improves the Treg/Th17 ratio in patients with RA, and alleviates disease activity with a favorable safety profile (65, 66). For example, Wang et al. demonstrated that refractory RA patients exhibited absolute CD4+ Treg depletion rather than Th17 expansion, and that low-dose IL-2 therapy (0.5 million IU, subcutaneously for 5 days) selectively restored Treg numbers, rebalanced Th17/Treg ratios, and induced clinical remission without adverse effects (65). Yan et al. demonstrated that difficult-to-treat (D2T) RA patients exhibited significant reductions in circulating T cells, CD4+ T cells, and particularly Tregs compared to healthy controls and treatment-responsive RA patients, resulting in elevated Th17/Treg ratios. In their study of 1, 042 RA patients and 339 healthy controls, low-dose IL-2 therapy (0.5 MIU daily, subcutaneously for 5 days) effectively expanded Treg populations across all RA groups without adverse effects, suggesting a unique lymphocyte depletionn phenotype in D2T RA amenable to IL-2 intervention (66). However, large-scale trials with long-term follow-up data remain limited.

##### 2.2.1.4 MS

In randomized, double-blind, placebo-controlled trials involving patients with MS, IL-2 therapy elicited modest and delayed Treg expansion, with some patients demonstrating elevated Treg frequencies and enhanced activation phenotypes; however, the response was relatively attenuated compared to that observed in other autoimmune diseases, and clinical benefits did not reach statistical significance (67). Novel IL-2 variants, such as efavaleukin alfa and MK-6194, have demonstrated more robust Treg expansion and improved safety profiles in diseases including SLE, with certain candidates advancing into early-phase clinical investigations for MS-related indications (68, 69). Several approved MS therapeutics (including siponimod, cladribine, and dimethyl fumarate) can indirectly promote Treg expansion or functional enhancement. For instance, siponimod treatment in secondary progressive MS (SPMS) patients enriches Treg and regulatory B cell populations, facilitating immune tolerance (70); cladribine therapy increases Treg abundance in cerebrospinal fluid (CSF), suggesting modulation of the central nervous system immune microenvironment (71); dimethyl fumarate elevates Treg proportions and ameliorates inflammatory responses (72). Emerging strategies, such as combination therapy with low-dose IL-2 and Treg expansion, or co-administration of Tregs with mesenchymal stem cells, have demonstrated synergistic immunomodulatory and neuroprotective effects in preclinical animal models and early-phase clinical studies (73).

##### 2.2.1.5 Pemphigus and other autoimmune skin diseases

Pemphigus vulgaris and related pemphigoid diseases are driven by autoantibodies against intercellular or basement membrane antigens, and Tregs are often numerically or functionally deficient in this setting (74, 75). Early-phase trials have explored the use of polyclonal autologous Tregs, and disease-relevant, antigen-specific Tregs in pemphigus to suppress autoreactive B and T cell responses (76). NCT03239470 has completed its early-phase evaluation, reporting favorable safety and tolerability; while efficacy signals require confirmation in larger cohorts, these efforts establish the feasibility of antigen-specific Treg approaches in autoimmune dermatology and support further development of DSG3-specific Tregs or broader autoreactive-Treg products as a means to attenuate pathogenic autoantibody production and inflammation. Mechanistically, DSG3-directed Tregs are anticipated to engage autoreactive B cells and Th1/Th17 effector pathways, rebalancing the humoral and cellular arms of the disease without general immunosuppression (77, 78).

In fact, Treg deficiency and Th17/Treg dysregulation represent pivotal pathogenic mechanisms across various autoimmune dermatoses, including vitiligo, alopecia areata, psoriasis, and systemic sclerosis (79–81). Therapeutic strategies aimed at restoring Treg homeostasis—via cellular expansion, functional potentiation, or enhanced cutaneous recruitment—hold promise for inducing antigen-specific tolerance and controlling skin inflammation (13, 81, 82). Despite this mechanistic rationale, most Treg-based interventions remain investigational, and rigorous clinical validation is required to address critical issues of efficacy persistence, infectious and neoplastic complications, and dermatologic specificity.

##### 2.2.1.6 Inflammatory bowel disease

Inflammatory bowel disease (IBD) remains an area of active Treg investigation because mucosal tolerance is a central driver of disease activity. In Crohn’s disease, trials employing ovalbumin-specific Tregs (CATS29) demonstrated safety and potential clinical benefit, with completed phase I/IIa testing (NCT03185000) suggesting that antigen-directed Tregs can be delivered in a manner compatible with gut inflammation and may modulate mucosal immune responses (83, 84). Although these studies used model antigen constructs, they provide a critical proof-of-concept for the feasibility of tolerogenic Treg therapy in human intestinal inflammation. The next generation of trials is expected to employ Crohn’s disease–relevant antigen specificities or prospectively tailored allo- or autologous Tregs to enhance localization to inflamed gut tissue and to suppress pathogenic Th1/Th17–driven pathways.

##### 2.2.1.7 Autoimmune hepatitis and other autoimmune liver diseases

Autoimmune hepatitis (AIH) represents a prototypic liver-directed autoimmune condition in which Treg-based strategies have been evaluated to reduce reliance on systemic immunosuppression (85). Autologous Treg infusion in AIH has been studied in early-phase trials (including I/IIa cohorts) with immunosuppressant tapering as a key outcome (85). These studies indicate that ex vivo expanded Tregs can be administered safely and may contribute to improved disease control, though data on long-term durability and histological endpoints remain limited. The AIH experience aligns with a broader concept of employing tolerogenic Tregs to recalibrate hepatic immune responses while preserving host defense.

##### 2.2.1.8 Other autoimmune diseases with emerging treg approaches

Explorations in additional autoimmune conditions—such as autoimmune thyroiditis (Hashimoto’s thyroiditis and Graves’ disease), celiac disease, and certain autoimmune skin and mucosal disorders—have yielded encouraging signals that Treg-directed therapies can modulate organ-specific immunopathology. In thyroiditis and celiac disease, preclinical data support Treg induction and functional stabilization as a means to dampen tissue-specific autoreactivity and restore mucosal or organ tolerance (86). Early clinical efforts in these diseases emphasize safety and tolerability, with expanding interest in antigen-specific Tregs and low-dose IL-2–mediated Treg enrichment as enabling strategies for disease-modifying responses (87). While larger, disease-specific trials are still needed to define efficacy endpoints, the accumulated data across autoimmune diseases consistently point to several shared principles: restoration of Treg numbers and function, enhancement of Treg stability in inflammatory milieus, and preferential expansion of regulatory compared with effector T cell subsets.

#### 2.2.2 Transplantation medicine

Tregs play a pivotal role in maintaining immune homeostasis and promoting tolerance in transplantation medicine (88). These cells suppress alloreactive immune responses, thereby reducing the risk of graft rejection and potentially minimizing the need for long-term immunosuppression (89, 90). Preclinical studies have demonstrated their efficacy in controlling acute and chronic rejection, highlighting their therapeutic potential for inducing operational tolerance in transplant recipients (91).

Clinical translation of Treg-based therapies in transplantation medicine has progressed significantly, with several Phase I and II trials investigating their safety and efficacy. Polyclonal Tregs, expanded ex vivo from recipient or donor sources, have been administered in settings such as solid organ transplantation and hematopoietic stem cell transplantation (HSCT) to prevent graft-versus-host disease (GVHD) (91, 92). For instance, early-phase trials have utilized ex vivo-expanded CD4+CD25+Foxp3+ Tregs, showing promising results in reducing immunosuppressive drug requirements and improving graft survival (89, 91).

Ongoing clinical efforts also focus on optimizing Treg manufacturing and delivery. For example, trials have investigated the use of donor-derived Tregs in liver and kidney transplantation, with preliminary evidence suggesting improved tolerance induction and reduced rejection episodes (90, 93). Future directions include leveraging metabolic programming to enhance Treg functionality *in vivo*, as well as combining Treg therapy with tolerogenic protocols such as costimulation blockade. While clinical outcomes are still evolving, Treg-based interventions represent a transformative approach toward achieving durable transplantation tolerance.

#### 2.2.3 Other frontiers in treg therapy

Beyond the well-established domains of autoimmunity and transplantation, Treg–based approaches are being explored in a broader range of non-malignant conditions, reflecting a growing appreciation for their roles in tissue homeostasis, repair, and systemic metabolism. In allergic diseases and airway inflammation, Tregs restrain allergen-driven Th2 responses and eosinophilic inflammation through cytokines such as IL-10 and TGF-β, contributing to mucosal tolerance and reduced airway hyperreactivity (94). Preclinical models of asthma and allergic rhinitis show that augmentation or adoptive transfer of Tregs can dampen local inflammation, while early human studies leveraging low-dose IL-2 to expand or stabilize Tregs indicate safety and potential biomarker improvements, underscoring the feasibility of organ-specific regulatory strategies; nonetheless, achieving efficient trafficking to the airway mucosa and preserving Treg stability in a Th2-biased environment remain critical challenges (95, 96).

In tissue repair and regenerative immunity, Tregs contribute to healing processes through mediators such as amphiregulin and interactions with resident stem or progenitor cells, with preclinical evidence showing accelerated wound repair and reduced fibrotic remodeling in various organ systems; translating these findings requires precise control of timing and localization to maximize repair while preserving antimicrobial defenses, with delivery modalities ranging from local injections to biomaterial-assisted recruitment and tissue-homing engineering (97, 98).

In immunometabolic diseases, Tregs modulate inflammatory tone and insulin sensitivity within adipose tissue, offering a mechanistic basis for treating metabolic syndrome and obesity-related inflammation; while animal models document improved metabolic outcomes upon Treg enhancement, human data remain preliminary, and strategies to selectively enrich Tregs in metabolic depots without inducing systemic immunosuppression are actively being explored (99).

The neuroinflammatory and CNS injury frontier draws on evidence that Tregs can modulate microglial activation and downstream inflammatory cascades, potentially limiting secondary injury and aiding recovery after stroke, traumatic brain injury, or spinal cord injury; however, crossing the blood–brain barrier, sustaining regulatory function in the CNS milieu, and balancing neuroprotection with adequate host defense are central obstacles that guide the design of delivery strategies and patient selection in early clinical work (100, 101).

In dermatology and mucosal health beyond autoimmune skin diseases, the skin and mucosal surfaces house specialized Treg populations that regulate barrier integrity and local immunity; preclinical studies suggest that enhancing regulatory networks can attenuate barrier-related inflammation and promote healing, with early clinical signals in related inflammatory conditions indicating tolerability and potential benefit, pointing to the value of local delivery and integration with barrier-restoration therapies to optimize outcomes (102).

Finally, other organ-system considerations and rare diseases comprise an expanding arena in which chronic inflammation, fibrosis, or remodeling may be modulated by Tregs; preclinical findings across liver, lung, and heart contexts hint at potential benefit, while early clinical exploration remains cautious and hypothesis-generating, underscoring the need for disease-specific endpoints, robust biomarkers, and long-term safety surveillance (14, 103).

Taken together, these frontier areas reflect a broadening recognition that Tregs can support therapeutic objectives beyond classical autoimmunity and transplantation, but progress will depend on rigorous, multidisciplinary studies that define optimal cell products, dosing regimens, routes of administration, and combinatorial strategies that maximize tissue-specific benefits while preserving systemic immune competence.

### 2.3 Future treatment directions

Future advances in Treg therapy for non-malignant diseases should build on mechanistic insight and translational progress to deliver disease-focused products that are safe, scalable, and monitorable in real-world clinical settings. The overarching goal is to translate fundamental Treg biology into precise, tissue-tolerant interventions whose effects can be rigorously evaluated in well-designed trials across autoimmune diseases and transplantation contexts. This trajectory will require harmonizing cell engineering, tissue targeting, dosing regimens, manufacturing pipelines, and immunomonitoring.

#### 2.3.1 Engineering tregs for precision tolerance

A central pillar of precision Treg therapy is the generation of antigen-specific regulatory cells, such as CAR-Tregs and TCR-Tregs, which can direct immunosuppressive activity to disease-relevant antigens while reducing systemic immunosuppression (104). Achieving a favorable safety profile necessitates careful balancing of potent local tolerance with preservation of host defense and avoidance of off-target effects. Enhancing the stability and suppressive phenotype of Tregs in inflammatory environments is critical; strategies that promote FOXP3 stability and durable epigenetic imprinting, including maintenance of TSDR demethylation and modulation of metabolic programs, are actively explored to sustain regulatory function (32, 105). Manufacturing practicality also guides design choices: non-viral engineering approaches, including mRNA-based methods and transposon systems, offer potential reductions in manufacturing complexity and regulatory burden, provided that efficacy and genomic safety are thoroughly validated (106, 107). Beyond genetic modification, advances in bioengineering, such as synthetic biology constructs that provide tunable co-stimulation or conditional regulatory circuits, hold promise for boosting potency while minimizing risk. Equally important is embedding tissue-homing features within engineered Tregs to promote localized immunoregulation at sites of inflammation or injury.

#### 2.3.2 Targeting tissue localization and delivery

Effective tissue targeting requires aligning Treg migratory capacity with the organ or tissue involved in disease. Engineering expression of disease-relevant chemokine receptors and adhesion molecules can enhance selective trafficking to affected sites, thereby improving efficacy and reducing systemic immunosuppression (108). Local administration strategies, including intralesional or organ-specific injections and the use of biomaterial platforms such as scaffolds or hydrogel systems, may concentrate Tregs at pathological sites, potentially increasing persistence and function (109). Comprehensive *in vivo* tracking, through imaging modalities or safe labeling approaches, supports safety and dosing decisions by delineating biodistribution and persistence over time. Taken together, optimized localization strategies should be designed to maximize regulatory impact where it is most needed while maintaining overall immune competence.

#### 2.3.3 Regimen design and combination strategies

The clinical implementation of Treg therapy depends critically on cell source selection and manufacturing processes. Polyclonal Tregs are most commonly derived from autologous peripheral blood mononuclear cells (PBMCs), though allogeneic sources—including umbilical cord blood and third-party donors—offer potential “off-the-shelf” advantages, albeit with considerations regarding HLA matching and persistence (110). Enrichment strategies typically rely on fluorescence-activated cell sorting (FACS) or magnetic bead-based isolation targeting CD4^+^CD25^+^CD127^low^ cells, with some protocols incorporating additional markers such as CD45RA to select naïve Treg subsets with superior stability (111). Ex vivo expansion is generally achieved through anti-CD3/CD28 bead stimulation supplemented with high-dose IL-2, with or without rapamycin to enhance FOXP3 stability and suppress effector T cell outgrowth (112). These manufacturing variables—including expansion duration, feeder cell use, and culture conditions—substantially influence product purity, FOXP3 demethylation status, suppressive potency, and phenotypic stability, contributing to heterogeneity across clinical studies and complicating cross-trial comparisons (113).

Beyond manufacturing considerations, Treg therapy will likely be most effective when integrated into tolerogenic regimens that sustain regulatory activity without unduly compromising host defense (114). Co-administration with low-dose IL-2 or other tolerogenic signals can support the expansion and maintenance of regulatory populations, yet requires careful timing and dose optimization to preserve effector responses necessary for pathogen control and anti-tumor surveillance (115, 116). Combination strategies that pair Tregs with other immunomodulatory modalities—such as B cell–targeted therapies, tolerogenic dendritic cells, or mesenchymal stromal cells—may yield synergistic benefits by simultaneously tempering multiple arms of the immune response (117, 118). Regimens should be disease-specific, reflecting underlying biology such as organ involvement, autoantigen repertoires, and inflammatory milieu, and should incorporate adaptive monitoring to adjust cell dosing and supportive therapies. Biomarker-guided personalization, leveraging flow cytometry, single-cell analyses, and functional assays, can inform patient selection, product choice (polyclonal versus antigen-specific), manufacturing protocol optimization, and treatment cadence, facilitating a move toward precision medicine in Treg-based interventions.

## 3 Challenges and research priorities

A major challenge in translating Treg therapies lies in the intrinsic heterogeneity and contextual stability of Treg subsets across tissues and diseases. Understanding how to preserve and direct regulatory phenotypes within diverse inflammatory milieus is essential, and will benefit from multi-omics and single-cell approaches to guide product design and patient selection. Crucially, the potent immunosuppressive capacity that makes Tregs therapeutically attractive also raises significant safety concerns. Pan-immunosuppression may impair host defense against pathogens, potentially leading to reactivation of latent infections such as cytomegalovirus (CMV), Epstein-Barr virus (EBV), or tuberculosis, and may theoretically compromise tumor immunosurveillance. Additionally, Treg plasticity poses a distinct risk: under certain inflammatory conditions, particularly those rich in IL-6 and IL-1β, Tregs may lose FOXP3 expression and convert into pathogenic effector phenotypes, including Th17-like or Th1-like cells, paradoxically exacerbating tissue inflammation rather than suppressing it. These phenotypic shifts have been documented in preclinical models and warrant careful monitoring in clinical settings. Translational barriers, including manufacturing scale-up, cost, regulatory scrutiny, and the need for comprehensive long-term safety data, must be addressed through investment in GMP-grade workflows, non-viral engineering platforms, and standardized potency and identity assays. While randomized controlled trials remain the gold standard for establishing efficacy, several autoimmune and transplantation studies have reported encouraging safety signals and clinical improvements in single-arm designs; these findings, albeit limited, provide important feasibility data and help shape the design of future multicenter, controlled studies. The field would benefit from biomarker development to enable pharmacodynamic and pharmacokinetic assessment, including Treg frequency and functionality, FOXP3/TSDR status, cytokine signatures, and tissue infiltration patterns, as well as predictive markers for response and adverse events. Finally, a proactive safety framework that anticipates risks of generalized immunosuppression, latent pathogen reactivation, or unintended pro-inflammatory shifts due to Treg instability should be integrated into product development from the outset.

## 4 Conclusion

Regulatory T cell therapy holds tangible promise for non-malignant diseases by restoring immune tolerance while preserving host defense. Realizing this potential will require disease-focused Treg products that combine antigen specificity with targeted tissue delivery, underpinned by rigorous manufacturing standards, comprehensive safety oversight, and dynamic immunomonitoring. Engineered Tregs, including CAR-Tregs and TCR-Tregs, together with rational combination regimens and tolerogenic backbones, are likely to define the near-term clinical trajectory. Progress will depend on well-designed multicenter trials, standardized endpoints, and robust biomarkers that enable patient stratification and adaptive therapy. Through iterative advances in cell engineering, delivery strategies, and translational science, Treg-based therapy can mature from an experimental modality into a mainstream clinical option that improves outcomes across autoimmune diseases and transplantation.

## References

[1] Bayati F Mohammadi M Valadi M Jamshidi S Foma AM Sharif-Paghaleh E . The therapeutic potential of regulatory T cells: challenges and opportunities. Front Immunol. (2020) 11:585819. doi: 10.3389/fimmu.2020.585819. PMID: 33519807 PMC7844143

[2] Georgiev P Benamar M Han S Haigis MC Sharpe AH Chatila TA . Regulatory T cells in dominant immunologic tolerance. J Allergy Clin Immunol. (2024) 153:28–41. doi: 10.1016/j.jaci.2023.09.025. PMID: 37778472 PMC10842646

[3] Benamar M Chen Q Martinez-Blanco M Chatila TA . Regulatory T cells in allergic inflammation. Semin Immunol. (2023) 70:101847. doi: 10.1016/j.smim.2023.101847. PMID: 37837939 PMC10842049

[4] Ghobadinezhad F Ebrahimi N Mozaffari F Moradi N Beiranvand S Pournazari M . The emerging role of regulatory cell-based therapy in autoimmune disease. Front Immunol. (2022) 13:1075813. doi: 10.3389/fimmu.2022.1075813. PMID: 36591309 PMC9795194

[5] Singh RP Bischoff DS Hahn BH . CD8+ T regulatory cells in lupus. Rheumatol Immunol Res. (2021) 2:147–56. doi: 10.2478/rir-2021-0021. PMID: 35880241 PMC9242525

[6] Rojas M Heuer LS Zhang W Chen Y-G Ridgway WM . The long and winding road: from mouse linkage studies to a novel human therapeutic pathway in type 1 diabetes. Front Immunol. (2022) 13:918837. doi: 10.3389/fimmu.2022.918837. PMID: 35935980 PMC9353112

[7] Lui PP Cho I Ali N . Tissue regulatory T cells. Immunology. (2020) 161:4–17. doi: 10.1111/imm.13208. PMID: 32463116 PMC7450170

[8] Muñoz-Rojas AR Mathis D . Tissue regulatory T cells: regulatory chameleons. Nat Rev Immunol. (2021) 21:597–611. doi: 10.1038/s41577-021-00519-w. PMID: 33772242 PMC8403160

[9] He J Ou K Schmueck-Henneresse M Specker E Paul J Nazare M . Generation of regulatory T cells from human memory CD4+T cells by upregulation of naked cuticle homolog 2. Eur J Immunol. (2025) 55:e70018. doi: 10.1002/eji.70018. PMID: 40762106 PMC12322872

[10] McCallion O Bilici M Hester J Issa F . Regulatory T-cell therapy approaches. Clin Exp Immunol. (2023) 211:96–107. doi: 10.1093/cei/uxac078. PMID: 35960852 PMC10019137

[11] Chen X Yang X Yuan P Jin R Bao L Qiu X . Modular immune-homeostatic microparticles promote immune tolerance in mouse autoimmune models. Sci Transl Med. (2021) 13:eaaw9668. doi: 10.1126/scitranslmed.aaw9668. PMID: 33692135 PMC12230986

[12] Lu Y Wang Y Ruan T Wang Y Ju L Zhou M . Immunometabolism of Tregs: mechanisms, adaptability, and therapeutic implications in diseases. Front Immunol. (2025) 16:1536020. doi: 10.3389/fimmu.2025.1536020. PMID: 39917294 PMC11798928

[13] Jugder B-E Park E Du L Jawale C Popov N Guo Z . Tissue-specific roles of regulatory T cells: mechanisms of suppression and beyond along with emerging therapeutic insights in autoimmune indications. Front Immunol. (2025) 16:1650451. doi: 10.3389/fimmu.2025.1650451. PMID: 40933988 PMC12417136

[14] Savage PA Klawon DEJ Miller CH . Regulatory T cell development. Annu Rev Immunol. (2020) 38:421–53. doi: 10.1146/annurev-immunol-100219-020937. PMID: 31990619

[15] Ødum N . Anti-regulatory T cells are natural regulatory effector T cells. Cell Stress. (2019) 3:310–1. doi: 10.15698/cst2019.10.199. PMID: 31680691 PMC6789433

[16] Yu Y Bai H Wu F Chen J Li B Li Y . Tissue adaptation of regulatory T cells in adipose tissue. Eur J Immunol. (2022) 52:1898–908. doi: 10.1002/eji.202149527. PMID: 36369886

[17] Miragaia RJ Gomes T Chomka A Jardine L Riedel A Hegazy AN . Single-cell transcriptomics of regulatory T cells reveals trajectories of tissue adaptation. Immunity. (2019) 50:493–504.e7. doi: 10.1016/j.immuni.2019.01.001. PMID: 30737144 PMC6382439

[18] Zong Y Deng K Chong WP . Regulation of Treg cells by cytokine signaling and co-stimulatory molecules. Front Immunol. (2024) 15:1387975. doi: 10.3389/fimmu.2024.1387975. PMID: 38807592 PMC11131382

[19] Toomer KH Malek TR . Cytokine signaling in the development and homeostasis of regulatory T cells. Cold Spring Harb Perspect Biol. (2018) 10:a028597. doi: 10.1101/cshperspect.a028597. PMID: 28620098 PMC5830895

[20] Lužnik Z Anchouche S Dana R Yin J . Regulatory T cells in angiogenesis. J Immunol. (2020) 205:2557–65. doi: 10.4049/jimmunol.2000574. PMID: 33168598 PMC7664842

[21] Tan BJY Ono M Satou Y . Single-cell transcriptome analysis of Treg. Methods Mol Biol. (2023) 2559:259–78. doi: 10.1007/978-1-0716-2647-4_17. PMID: 36180638

[22] Wing K Onishi Y Prieto-Martin P Yamaguchi T Miyara M Fehervari Z . CTLA-4 control over Foxp3+ regulatory T cell function. Science. (2008) 322:271–5. doi: 10.1126/science.1160062. PMID: 18845758

[23] Chinen T Kannan AK Levine AG Fan X Klein U Zheng Y . An essential role for the IL-2 receptor in Treg cell function. Nat Immunol. (2016) 17:1322–33. doi: 10.1038/ni.3540. PMID: 27595233 PMC5071159

[24] Bolivar-Wagers S Larson JH Jin S Blazar BR . Cytolytic CD4+ and CD8+ regulatory T-cells and implications for developing immunotherapies to combat graft-versus-host disease. Front Immunol. (2022) 13:864748. doi: 10.3389/fimmu.2022.864748. PMID: 35493508 PMC9040077

[25] Deaglio S Dwyer KM Gao W Friedman D Usheva A Erat A . Adenosine generation catalyzed by CD39 and CD73 expressed on regulatory T cells mediates immune suppression. J Exp Med. (2007) 204:1257–65. doi: 10.1084/jem.20062512. PMID: 17502665 PMC2118603

[26] Mathur AN Zirak B Boothby IC Tan M Cohen JN Mauro TM . Treg-cell control of a CXCL5-IL-17 inflammatory axis promotes hair-follicle-stem-cell differentiation during skin-barrier repair. Immunity. (2019) 50:655–667.e4. doi: 10.1016/j.immuni.2019.02.013. PMID: 30893588 PMC6507428

[27] Collison LW Chaturvedi V Henderson AL Giacomin PR Guy C Bankoti J . IL-35-mediated induction of a potent regulatory T cell population. Nat Immunol. (2010) 11:1093–101. doi: 10.1038/ni.1952. PMID: 20953201 PMC3008395

[28] Levings MK Sangregorio R Sartirana C Moschin AL Battaglia M Orban PC . Human CD25+CD4+ T suppressor cell clones produce transforming growth factor beta, but not interleukin 10, and are distinct from type 1 T regulatory cells. J Exp Med. (2002) 196:1335–46. doi: 10.1084/jem.20021139. PMID: 12438424 PMC2193983

[29] Couper KN Blount DG Riley EM . IL-10: the master regulator of immunity to infection. J Immunol. (2008) 180:5771–7. doi: 10.4049/jimmunol.180.9.5771. PMID: 18424693

[30] Ningoo M Fueyo-González F Gisbert-Vilanova C Espinar-Barranco L Marjanovic N Fribourg M . Interferon-β and interleukin-6 exert opposing effects on Foxp3 acetylation to control regulatory T cell induction. Front Immunol. (2025) 16:1593931. doi: 10.3389/fimmu.2025.1593931. PMID: 40519931 PMC12162494

[31] Göschl L Scheinecker C Bonelli M . Treg cells in autoimmunity: from identification to Treg-based therapies. Semin Immunopathol. (2019) 41:301–14. doi: 10.1007/s00281-019-00741-8. PMID: 30953162

[32] Arroyo-Olarte RD Flores-Castelán JC Armas-López L Escobedo G Terrazas LI Ávila-Moreno F . Targeted demethylation of FOXP3-TSDR enhances the suppressive capacity of STAT6-deficient inducible T regulatory cells. Inflammation. (2024) 47:2159–72. doi: 10.1007/s10753-024-02031-4. PMID: 38700792 PMC11606997

[33] Medof ME Rieder SA Shevach EM . Disabled C3ar1/C5ar1 signaling in Foxp3+ T regulatory cells leads to TSDR demethylation and long-term stability. J Immunol. (2023) 211:1359–66. doi: 10.4049/jimmunol.2300184. PMID: 37756526 PMC10591991

[34] Ngalamika O Liang G Zhao M Yu X Yang Y Yin H . Peripheral whole blood FOXP3 TSDR methylation: a potential marker in severity assessment of autoimmune diseases and chronic infections. Immunol Invest. (2015) 44:126–36. doi: 10.3109/08820139.2014.938165. PMID: 25083793

[35] Matsuoka K Koreth J Kim HT Bascug G McDonough S Kawano Y . Low-dose interleukin-2 therapy restores regulatory T cell homeostasis in patients with chronic graft-versus-host disease. Sci Transl Med. (2013) 5:179ra43. doi: 10.1126/scitranslmed.3005265. PMID: 23552371 PMC3686517

[36] Tuomela K Salim K Levings MK . Eras of designer Tregs: harnessing synthetic biology for immune suppression. Immunol Rev. (2023) 320:250–67. doi: 10.1111/imr.13254. PMID: 37522861

[37] Hull CM Peakman M Tree TIM . Regulatory T cell dysfunction in type 1 diabetes: what’s broken and how can we fix it? Diabetologia. (2017) 60:1839–50. doi: 10.1007/s00125-017-4377-1. PMID: 28770318 PMC6448885

[38] Romano M Fanelli G Albany CJ Giganti G Lombardi G . Past, present, and future of regulatory T cell therapy in transplantation and autoimmunity. Front Immunol. (2019) 10:43. doi: 10.3389/fimmu.2019.00043. PMID: 30804926 PMC6371029

[39] Bender C Wiedeman AE Hu A Ylescupidez A Sietsema WK Herold KC . A phase 2 randomized trial with autologous polyclonal expanded regulatory T cells in children with new-onset type 1 diabetes. Sci Transl Med. (2024) 16:eadn2404. doi: 10.1126/scitranslmed.adn2404. PMID: 38718135

[40] Rosenzwajg M Salet R Lorenzon R Tchitchek N Roux A Bernard C . Low-dose IL-2 in children with recently diagnosed type 1 diabetes: a phase I/II randomised, double-blind, placebo-controlled, dose-finding study. Diabetologia. (2020) 63:1808–21. doi: 10.1007/s00125-020-05200-w. PMID: 32607749

[41] Liu Y-F Powrie J Arif S Yang JHM Williams E Khatri L . Immune and metabolic effects of antigen-specific immunotherapy using multiple β-cell peptides in type 1 diabetes. Diabetes. (2022) 71:722–32. doi: 10.2337/db21-0728. PMID: 35073398 PMC8965665

[42] Zieliński M Żalińska M Iwaszkiewicz-Grześ D Gliwiński M Hennig M Jaźwińska-Curyłło A . Combined therapy with CD4+ CD25highCD127- T regulatory cells and anti-CD20 antibody in recent-onset type 1 diabetes is superior to monotherapy: randomized phase I/II trial. Diabetes Obes Metab. (2022) 24:1534–43. doi: 10.1111/dom.14723. PMID: 35441440

[43] Zieliński M Sakowska J Iwaszkiewicz-Grześ D Gliwiński M Hennig M Żalińska M . PD-1 receptor (+) T cells are associated with the efficacy of the combined treatment with regulatory t cells and rituximab in type 1 diabetes children via regulatory t cells suppressive activity amelioration. Int Immunopharmacol. (2024) 132:111919. doi: 10.1016/j.intimp.2024.111919. PMID: 38554443

[44] Russell WE Bundy BN Anderson MS Cooney LA Gitelman SE Goland RS . Abatacept for delay of type 1 diabetes progression in stage 1 relatives at risk: a randomized, double-masked, controlled trial. Diabetes Care. (2023) 46:1005–13. doi: 10.2337/dc22-2200. PMID: 36920087 PMC10154649

[45] Marcovecchio ML Wicker LS Dunger DB Dutton SJ Kopijasz S Scudder C . Interleukin-2 therapy of autoimmunity in diabetes (ITAD): a phase 2, multicentre, double-blind, randomized, placebo-controlled trial. Wellcome Open Res. (2020) 5:49. doi: 10.12688/wellcomeopenres.15697.1. PMID: 32399500 PMC7194454

[46] Gregory JW Carter K Cheung WY Holland G Bowen-Morris J Luzio S . Phase II multicentre, double-blind, randomised trial of ustekinumab in adolescents with new-onset type 1 diabetes (USTEK1D): trial protocol. BMJ Open. (2021) 11:e049595. doi: 10.1136/bmjopen-2021-049595. PMID: 34663658 PMC8524290

[47] Gaglia JL Daley HL Bryant NK Ritz J Dong T Skyler JS . Novel autologous dendritic cell therapy AVT001 for type 1 diabetes. NEJM Evid. (2024) 3:EVIDoa2300238. doi: 10.1056/EVIDoa2300238. PMID: 38916421 PMC11697638

[48] Jacobsen LM Diggins K Blanchfield L McNichols J Perry DJ Brant J . Responders to low-dose ATG induce CD4+ T cell exhaustion in type 1 diabetes. JCI Insight. (2023) 8:e161812. doi: 10.1172/jci.insight.161812. PMID: 37432736 PMC10543726

[49] He J Zhang X Wei Y Sun X Chen Y Deng J . Low-dose interleukin-2 treatment selectively modulates CD4(+) T cell subsets in patients with systemic lupus erythematosus. Nat Med. (2016) 22:991–3. doi: 10.1038/nm.4148. PMID: 27500725

[50] Akbarzadeh R Riemekasten G Humrich JY . Low-dose interleukin-2 therapy: a promising targeted therapeutic approach for systemic lupus erythematosus. Curr Opin Rheumatol. (2023) 35:98–106. doi: 10.1097/BOR.0000000000000924. PMID: 36563007

[51] von Spee-Mayer C Siegert E Abdirama D Rose A Klaus A Alexander T . Low-dose interleukin-2 selectively corrects regulatory T cell defects in patients with systemic lupus erythematosus. Ann Rheum Dis. (2016) 75:1407–15. doi: 10.1136/annrheumdis-2015-207776. PMID: 26324847

[52] Farooq A Trehan S Singh G Arora N Mehta T Jain P . A comprehensive review of low-dose interleukin-2 (IL-2) therapy for systemic lupus erythematosus: mechanisms, efficacy, and clinical applications. Cureus. (2024) 16:e68748. doi: 10.7759/cureus.68748. PMID: 39371877 PMC11455659

[53] Humrich JY Cacoub P Rosenzwajg M Pitoiset F Pham HP Guidoux J . Low-dose interleukin-2 therapy in active systemic lupus erythematosus (LUPIL-2): a multicentre, double-blind, randomised and placebo-controlled phase II trial. Ann Rheum Dis. (2022) 81:1685–94. doi: 10.1136/ard-2022-222501. PMID: 35973803

[54] Fanton C Furie R Chindalore V Levin R Diab I Dixit N . Selective expansion of regulatory T cells by NKTR-358 in healthy volunteers and patients with systemic lupus erythematosus. J Trans Autoimmun. (2022) 5:100152. doi: 10.1016/j.jtauto.2022.100152. PMID: 35517914 PMC9062472

[55] Sarkar N Hu X Tchao N Furie R Kivitz A Cohen S . OP0140 regulatory T cell defects in SLE and therapy with a novel IL-2 mutein: phase 1 clinical results with efavaleukin alfa. Ann Rheum Dis. (2023) 82:92–3. doi: 10.1136/annrheumdis-2023-eular.369

[56] Kim ND Valiyil R Pangan AL Werth VP Petri M Furie R . Design of a phase 2a, multicenter, randomized, double-blind, placebo-controlled trial of Mk-6194, an interleukin-2 mutein, in adult participants with systemic lupus erythematosus. J Rheumatol. (2025) 52:245–6. doi: 10.3899/jrheum.2025-0390.PV264

[57] Eggenhuizen PJ Cheong RMY Lo C Chang J Ng BH Ting YT . Smith-specific regulatory T cells halt the progression of lupus nephritis. Nat Commun. (2024) 15:899. doi: 10.1038/s41467-024-45056-x. PMID: 38321013 PMC10847119

[58] Dall’Era M Pauli ML Remedios K Taravati K Sandova PM Putnam AL . Adoptive Treg cell therapy in a patient with systemic lupus erythematosus. Arthritis Rheumatol. (2019) 71:431–40. doi: 10.1002/art.40737. PMID: 30277008 PMC6447289

[59] Doglio M Ugolini A Bercher-Brayer C . Regulatory T cells expressing CD19-targeted chimeric antigen receptor restore homeostasis in Systemic Lupus Erythematosus. Nat Commun (2024) 15: 2542. doi: 10.1038/s41467-024-46448-9 38538608 PMC10973480

[60] Chen Y Tao T Wang W Yang B Cha X . Dihydroartemisinin attenuated the symptoms of mice model of systemic lupus erythematosus by restoring the Treg/Th17 balance. Clin Exp Pharmacol Physiol. (2021) 48:626–33. doi: 10.1111/1440-1681.13461. PMID: 33469936

[61] Zhang J Chang L Sun Y Qin M Wang X Guo Y . Disabled-2 overexpression mediates immune suppression in systemic lupus erythematosus by modulating Treg/Th17 cell differentiation. Clin Exp Pharmacol Physiol. (2022) 49:596–607. doi: 10.1111/1440-1681.13630. PMID: 35108421

[62] Ji J Liang Q He Q Chen T Feng G Guo H . Overexpression of miR-20a-5p in mesenchymal stem cell derived-exosomes from systemic lupus erythematosus patients restored therapeutic effect and Treg immune regulation. Eur J Pharmacol. (2024) 979:176862. doi: 10.1016/j.ejphar.2024.176862. PMID: 39068974

[63] Li S Wang H Wu H Chang X . Therapeutic effect of exogenous regulatory T cells on collagen-induced arthritis and rheumatoid arthritis. Cell Transplant. (2020) 29:96368972095413. doi: 10.1177/0963689720954134. PMID: 32990025 PMC7784507

[64] Chmiel J Stasiak M Skrzypkowska M Samson L Łuczkiewicz P Trzonkowski P . Regulatory T lymphocytes as a treatment method for rheumatoid arthritis – superiority of allogeneic to autologous cells. Heliyon. (2024) 10(17):e36512. doi: 10.1016/j.heliyon.2024.e36512. PMID: 39319132 PMC11419861

[65] Wang J Zhang S-X Chang J-S Cheng T Jiang X-J Su Q-Y . Low-dose IL-2 improved clinical symptoms by restoring reduced regulatory T cells in patients with refractory rheumatoid arthritis: a randomized controlled trial. Front Immunol. (2022) 13:947341. doi: 10.3389/fimmu.2022.947341. PMID: 36524114 PMC9744779

[66] Yan H Zi X Yan H Zhang X Bai J Gao C . The absolute number of circulating Treg cells is reduced in difficult-to-treat RA patients and is ameliorated by low-dose IL-2. Front Immunol. (2025) 16:1522893. doi: 10.3389/fimmu.2025.1522893. PMID: 39981233 PMC11839615

[67] Louapre C Rosenzwajg M Golse M Roux A Pitoiset F Adda L . A randomized double-blind placebo-controlled trial of low-dose interleukin-2 in relapsing-remitting multiple sclerosis. J Neurol. (2023) 270:4403–14. doi: 10.1007/s00415-023-11690-6. PMID: 37245191

[68] Scheid JF Cunningham-Bussel K Kim N Agarwal S Nieddu G Cote J . Safety, pharmacokinetics, and pharmacodynamics of MK-6194, an IL-2 mutein designed to selectively activate regulatory T cells: single ascending dose and multiple ascending dose trial data. Immunohorizons. (2025) 9:vlaf005. doi: 10.1093/immhor/vlaf005. PMID: 40139976 PMC11945303

[69] Tchao N Sarkar N Hu X Zhang R Milmont C Shi Jin Y . AB0432 efavaleukin alfa, a novel IL-2 mutein, selectively expands regulatory T cells in patients with SLE: final results of a phase 1B multiple ascending dose study. Ann Rheum Dis. (2022) 81:1343–4. doi: 10.1136/annrheumdis-2022-eular.2244

[70] Wu Q Mills EA Wang Q Dowling CA Fisher C Kirch B . Siponimod enriches regulatory T and B lymphocytes in secondary progressive multiple sclerosis. JCI Insight. (2020) 5:e134251, 134251. doi: 10.1172/jci.insight.134251. PMID: 31935197 PMC7098784

[71] Schroeter CB Rolfes L Gothan KSS Gruchot J Herrmann AM Bock S . Cladribine treatment improves cortical network functionality in a mouse model of autoimmune encephalomyelitis. J Neuroinflamm. (2022) 19:270. doi: 10.1186/s12974-022-02588-7. PMID: 36348455 PMC9641831

[72] Vucic S Ryder J Mekhael L Rd H Mathers S Needham M . Phase 2 randomized placebo controlled double blind study to assess the efficacy and safety of tecfidera in patients with amyotrophic lateral sclerosis (TEALS Study): study protocol clinical trial (SPIRIT compliant). Med (Baltimore). (2020) 99:e18904. doi: 10.1097/MD.0000000000018904. PMID: 32028398 PMC7015658

[73] Sadeghnejad A Pazoki A Yazdanpanah E Esmaeili S-A Yousefi B Sadighi-Moghaddam B . Exploring the role of mesenchymal stem cells in modulating immune responses via Treg and Th2 cell activation: insights from mouse model of multiple sclerosis. APMIS. (2024) 132:888–99. doi: 10.1111/apm.13456. PMID: 39030955

[74] Fang H Li Q Wang G . The role of T cells in pemphigus vulgaris and bullous pemphigoid. Autoimmun Rev. (2020) 19:102661. doi: 10.1016/j.autrev.2020.102661. PMID: 32942041

[75] Muramatsu K Zheng M Yoshimoto N Ito T Ujiie I Iwata H . Regulatory T cell subsets in bullous pemphigoid and dipeptidyl peptidase-4 inhibitor-associated bullous pemphigoid. J Dermatol Sci. (2020) 100:23–30. doi: 10.1016/j.jdermsci.2020.08.004. PMID: 32843228

[76] Lee J Lundgren DK Mao X Manfredo-Vieira S Nunez-Cruz S Williams EF . Antigen-specific B cell depletion for precision therapy of mucosal pemphigus vulgaris. J Clin Invest. (2020) 130:6317–24. doi: 10.1172/JCI138416. PMID: 32817591 PMC7685721

[77] Goswami TK Singh M Dhawan M Mitra S Emran TB Rabaan AA . Regulatory T cells (Tregs) and their therapeutic potential against autoimmune disorders - advances and challenges. Hum Vaccin Immunother. (2022) 18:2035117. doi: 10.1080/21645515.2022.2035117. PMID: 35240914 PMC9009914

[78] Fisher MS Sennikov SV . T-regulatory cells for the treatment of autoimmune diseases. Front Immunol. (2025) 16:1511671. doi: 10.3389/fimmu.2025.1511671. PMID: 39967659 PMC11832489

[79] Jin S Wan S Xiong R Li Y Dong T Guan C . The role of regulatory T cells in vitiligo and therapeutic advances: a mini-review. Inflammation Res. (2024) 73:1311–32. doi: 10.1007/s00011-024-01900-w. PMID: 38839628

[80] Lerner G Nikolaou M Stoffel C Schmauch E Kündig T Passeron T . Regulatory T cell dysregulation in vitiligo: a meta-analysis and systematic review of immune mechanisms and therapeutic perspectives. Int J Dermatol. (2025) 64:2247–56. doi: 10.1111/ijd.17959. PMID: 40660423 PMC12605810

[81] Mukhatayev Z Ostapchuk YO Fang D Le Poole IC . Engineered antigen-specific regulatory T cells for autoimmune skin conditions. Autoimmun Rev. (2021) 20:102761. doi: 10.1016/j.autrev.2021.102761. PMID: 33476816 PMC9285649

[82] Selck C Dominguez-Villar M . Antigen-specific regulatory T cell therapy in autoimmune diseases and transplantation. Front Immunol. (2021) 12:661875. doi: 10.3389/fimmu.2021.661875. PMID: 34054826 PMC8160309

[83] Clough JN Omer OS Tasker S Lord GM Irving PM . Regulatory T-cell therapy in Crohn’s disease: challenges and advances. Gut. (2020) 69:942–52. doi: 10.1136/gutjnl-2019-319850. PMID: 31980447 PMC7229901

[84] Desreumaux P Foussat A Allez M Beaugerie L Hébuterne X Bouhnik Y . Safety and efficacy of antigen-specific regulatory T-cell therapy for patients with refractory Crohn’s disease. Gastroenterology. (2012) 143:1207–1217.e2. doi: 10.1053/j.gastro.2012.07.116. PMID: 22885333

[85] Oo YH Ackrill S Cole R Jenkins L Anderson P Jeffery HC . Liver homing of clinical grade Tregs after therapeutic infusion in patients with autoimmune hepatitis. JHEP Rep. (2019) 1:286–96. doi: 10.1016/j.jhepr.2019.08.001. PMID: 32039380 PMC7001578

[86] Kustrimovic N Gallo D Piantanida E Bartalena L Lai A Zerbinati N . Regulatory T cells in the pathogenesis of Graves’ disease. Int J Mol Sci. (2023) 24:16432. doi: 10.3390/ijms242216432. PMID: 38003622 PMC10671795

[87] Rosenzwajg M Lorenzon R Cacoub P Pham HP Pitoiset F El Soufi K . Immunological and clinical effects of low-dose interleukin-2 across 11 autoimmune diseases in a single, open clinical trial. Ann Rheum Dis. (2019) 78:209–17. doi: 10.1136/annrheumdis-2018-214229. PMID: 30472651

[88] Wood KJ . Regulatory T cells in transplantation. Transplant Proc. (2011) 43:2135–6. doi: 10.1016/j.transproceed.2011.06.050. PMID: 21839214 PMC3202641

[89] Albert MH Anasetti C Yu X-Z . T regulatory cells as an immunotherapy for transplantation. Expert Opin Biol Ther. (2006) 6:315–24. doi: 10.1517/14712598.6.4.315. PMID: 16548760

[90] Khan MA . T regulatory cell mediated immunotherapy for solid organ transplantation: a clinical perspective. Mol Med. (2017) 22:892–904. doi: 10.2119/molmed.2016.00050. PMID: 27878210 PMC5319206

[91] McMurchy AN Bushell A Levings MK Wood KJ . Moving to tolerance: clinical application of T regulatory cells. Semin Immunol. (2011) 23:304–13. doi: 10.1016/j.smim.2011.04.001. PMID: 21620722 PMC3836227

[92] Lam AJ Hoeppli RE Levings MK . Harnessing advances in T regulatory cell biology for cellular therapy in transplantation. Transplantation. (2017) 101:2277–87. doi: 10.1097/TP.0000000000001757. PMID: 28376037

[93] Jiang G Yang H-R Wang L Wildey GM Fung J Qian S . Hepatic stellate cells preferentially expand allogeneic CD4+ CD25+ FoxP3+ regulatory T cells in an IL-2-dependent manner. Transplantation. (2008) 86:1492–502. doi: 10.1097/TP.0b013e31818bfd13. PMID: 19077880 PMC2888269

[94] Zhang J Zou Y Chen L Xu Q Wang Y Xie M . Regulatory T cells, a viable target against airway allergic inflammatory responses in asthma. Front Immunol. (2022) 13:902318. doi: 10.3389/fimmu.2022.902318. PMID: 35757774 PMC9226301

[95] Rosenzwajg M Gherasim A Dietsch F Beck M Domis N Lorenzon R . Low-dose IL-2 in birch pollen allergy: a phase-2 randomized double-blind placebo-controlled trial. J Allergy Clin Immunol. (2025) 155:650–5. doi: 10.1016/j.jaci.2024.10.033. PMID: 39532189

[96] Skuljec J Chmielewski M Happle C Habener A Busse M Abken H . Chimeric antigen receptor-redirected regulatory T cells suppress experimental allergic airway inflammation, a model of asthma. Front Immunol. (2017) 8:1125. doi: 10.3389/fimmu.2017.01125. PMID: 28955341 PMC5600908

[97] Jie J Yao X Deng H Zhou Y Jiang X Dai X . Regulatory T cells in neurological disorders and tissue regeneration: Mechanisms of action and therapeutic potentials. Neural Regener Res. (2026) 21:1277–91. doi: 10.4103/NRR.NRR-D-24-01363. PMID: 40536993 PMC12407513

[98] Li J Tan J Martino MM Lui KO . Regulatory T-cells: Potential regulator of tissue repair and regeneration. Front Immunol. (2018) 9:585. doi: 10.3389/fimmu.2018.00585. PMID: 29662491 PMC5890151

[99] Elkins C Ye C Sivasami P Mulpur R Diaz-Saldana PP Peng A . Obesity reshapes regulatory T cells in the visceral adipose tissue by disrupting cellular cholesterol homeostasis. Sci Immunol. (2025) 10:eadl4909. doi: 10.1126/sciimmunol.adl4909. PMID: 39792637 PMC11786953

[100] Zhang C Li Y Yu Y Li Z Xu X Talifu Z . Impact of inflammation and Treg cell regulation on neuropathic pain in spinal cord injury: Mechanisms and therapeutic prospects. Front Immunol. (2024) 15:1334828. doi: 10.3389/fimmu.2024.1334828. PMID: 38348031 PMC10859493

[101] Liu R Li Y Wang Z Chen P Xie Y Qu W . Regulatory T cells promote functional recovery after spinal cord injury by alleviating microglia inflammation via STAT3 inhibition. CNS Neurosci Ther. (2023) 29:2129–44. doi: 10.1111/cns.14161. PMID: 36914969 PMC10352886

[102] Moreau JM Dhariwala MO Gouirand V Boda DP Boothby IC Lowe MM . Regulatory T cells promote innate inflammation after skin barrier breach via TGF-β activation. Sci Immunol. (2021) 6:eabg2329. doi: 10.1126/sciimmunol.abg2329. PMID: 34452925 PMC8958044

[103] Zhang M Zhang S . T cells in fibrosis and fibrotic diseases. Front Immunol. (2020) 11:1142. doi: 10.3389/fimmu.2020.01142. PMID: 32676074 PMC7333347

[104] Arjomandnejad M Kopec AL Keeler AM . CAR-T regulatory (CAR-Treg) cells: Engineering and applications. Biomedicines. (2022) 10:287. doi: 10.3390/biomedicines10020287. PMID: 35203496 PMC8869296

[105] Kressler C Gasparoni G Nordström K Hamo D Salhab A Dimitropoulos C . Targeted de-methylation of the FOXP3-TSDR is sufficient to induce physiological FOXP3 expression but not a functional Treg phenotype. Front Immunol. (2020) 11:609891. doi: 10.3389/fimmu.2020.609891. PMID: 33488615 PMC7817622

[106] Chen Z Ren A Li Y Shu J Wu J Huang H . mRNA-laden lipid nanoparticle-enabled humanized CD19 CAR-T-cell engineering for the eradication of leukaemic cells. Br J Haematol. (2025) 206:628–43. doi: 10.1111/bjh.19988. PMID: 39761676 PMC11829146

[107] Moretti A Ponzo M Nicolette CA Tcherepanova IY Biondi A Magnani CF . The past, present, and future of non-viral CAR T cells. Front Immunol. (2022) 13:867013. doi: 10.3389/fimmu.2022.867013. PMID: 35757746 PMC9218214

[108] Whilding LM Halim L Draper B Parente-Pereira AC Zabinski T Davies DM . CAR T-cells targeting the integrin αvβ6 and co-expressing the chemokine receptor CXCR2 demonstrate enhanced homing and efficacy against several solid Malignancies. Cancers (Basel). (2019) 11:674. doi: 10.3390/cancers11050674. PMID: 31091832 PMC6563120

[109] Weiden J Voerman D Dölen Y Das RK van Duffelen A Hammink R . Injectable biomimetic hydrogels as tools for efficient T cell expansion and delivery. Front Immunol. (2018) 9:2798. doi: 10.3389/fimmu.2018.02798. PMID: 30546367 PMC6279891

[110] Khan MJ Lee YJ Lee SY Chung H Nguyen-Phuong T Kim Y-H . Novel autologous regulatory T-cell therapy ameliorates DSS-induced colitis in humanized mice. Inflammation Bowel Dis. (2025) 31:2535–46. doi: 10.1093/ibd/izaf141. PMID: 40622253 PMC12456575

[111] Arroyo Hornero R Betts GJ Sawitzki B Vogt K Harden PN Wood KJ . CD45RA distinguishes CD4+CD25+CD127-/low TSDR demethylated regulatory T cell subpopulations with differential stability and susceptibility to tacrolimus-mediated inhibition of suppression. Transplantation. (2017) 101:302–9. doi: 10.1097/TP.0000000000001278. PMID: 28118317 PMC5265687

[112] Rossetti M Spreafico R Saidin S Chua C Moshref M Leong JY . Ex vivo-expanded but not *in vitro*-induced human regulatory T cells are candidates for cell therapy in autoimmune diseases thanks to stable demethylation of the FOXP3 regulatory T cell-specific demethylated region. J Immunol. (2015) 194:113–24. doi: 10.4049/jimmunol.1401145. PMID: 25452562 PMC4383769

[113] Wang H Song H Pham AV Cooper LJ Schulze JJ Olek S . Human LAP+GARP+FOXP3+ regulatory T cells attenuate xenogeneic graft versus host disease. Theranostics. (2019) 9:2315–24. doi: 10.7150/thno.30254. PMID: 31149046 PMC6531299

[114] Ferreira LMR Muller YD Bluestone JA Tang Q . Next-generation regulatory T cell therapy. Nat Rev Drug Discov. (2019) 18:749–69. doi: 10.1038/s41573-019-0041-4. PMID: 31541224 PMC7773144

[115] Ye C Brand D Zheng SG . Targeting IL-2: An unexpected effect in treating immunological diseases. Signal Transduct Target Ther. (2018) 3:2. doi: 10.1038/s41392-017-0002-5. PMID: 29527328 PMC5837126

[116] Rosenzwajg M Churlaud G Mallone R Six A Dérian N Chaara W . Low-dose interleukin-2 fosters a dose-dependent regulatory T cell tuned milieu in T1D patients. J Autoimmun. (2015) 58:48–58. doi: 10.1016/j.jaut.2015.01.001. PMID: 25634360 PMC8153751

[117] Liu J Liu Q Chen X . The immunomodulatory effects of mesenchymal stem cells on regulatory B cells. Front Immunol. (2020) 11:1843. doi: 10.3389/fimmu.2020.01843. PMID: 32922398 PMC7456948

[118] Negi N Griffin MD . Effects of mesenchymal stromal cells on regulatory T cells: Current understanding and clinical relevance. Stem Cells. (2020) 38:596–605. doi: 10.1002/stem.3151. PMID: 31995249 PMC7217190

